# Pre-arrest doxycycline protects donation after circulatory death kidneys

**DOI:** 10.1038/s41598-020-79440-6

**Published:** 2020-12-17

**Authors:** Michael Moser, Sarah Schmid, Katherine Sawicka, Tamalina Banerjee, Erick McNair, Jolanta Sawicka, Iwona Bil-Lula, Grzegorz Sawicki

**Affiliations:** 1 Department of Surgery, University of Saskatchewan, St. Paul’s Hospital, 1702 - 20th Street West, Saskatoon, SK S7M 0Z9 Canada; 2 Saskatchewan Renal Transplant Program, Saskatoon, SK Canada; 3 Department of Medicine, University of Toronto, Toronto, Canada; 4 Department of Pathology and Lab Medicine, University of Saskatchewan, Saskatoon, SK Canada; 5 Department of Anatomy, Physiology, and Pharmacology, College of Medicine, University of Saskatchewan, 107 Wiggins Road, Saskatoon, SK S7N 5E5 Canada; 6 Department of Clinical Chemistry, Medical University of Wroclaw, Wrocław, Poland

**Keywords:** Kidney, Renal replacement therapy, Translational research

## Abstract

Kidney injury during donation after circulatory determination of death (DCDD) includes warm ischemic (WI) injury from around the time of asystole, and cold ischemic (CI) injury during cold preservation. We have previously shown that Matrix Metalloproteinases (MMPs) are involved in CI injury and that Doxycycline (Doxy), an antibiotic and known MMP inhibitor, protects the transplant kidney during CI. The purpose of our study was to determine if Doxy given *before* asystole can also prevent injury during WI. A rat model of DCDD was used, including Control, Preemptive Doxy (45 mg/kg iv), and Preemptive and Perfusion (100 microM) Doxy groups. Thirty minutes after asystole, both kidneys were removed. The left kidney was perfused at 4 °C for 22 h, whereas the right was used to establish the degree of warm ischemic injury prior to cold preservation. MMP-2 in the perfusate was significantly reduced in both treatment groups [Control 43.7 ± 7.2 arbitrary units, versus Preemptive Doxy group 23.2 ± 5.5 (*p* = 0.03), and ‘Preemptive and Perfusion’ group 18.0 ± 5.6 (*p* = 0.02)]. Reductions in NGAL, LDH, and MMP-9 were also seen. Electron microscopy showed a marked reduction in mitochondrial injury scores in the treatment groups. Pre-arrest Doxy was associated with a reduction in injury markers and morphologic changes. Doxy may be a simple and safe means of protecting transplant kidneys from both WI and CI.

## Introduction

In an effort to increase the number of kidneys available for transplantation, transplant programs have revisited the use of kidneys from Donation after Circulatory Determination of Death (DCDD). Donation after neurological determination of death (DNDD) had been the only source of deceased donor organs for the vast majority of transplant programs since the late 1960s^1^. Unfortunately, DCDD donor kidneys are associated with a higher rate of delayed graft function, and reduced graft survival^2,3^. Whereas warm ischemia (WI) at the time of procurement is minimal in DNDD donation, there is considerable warm (37 °C) ischemia in DCDD-retrieved kidneys and this may account for the worse outcomes with these organs.

Matrix Metalloproteinases (MMPs) are a family of proteolytic enzymes that play important roles in a variety of physiological and pathological processes^4^. They are best known for the degradation of extracellular proteins and remodeling of the extracellular matrix (ECM) but also have intracellular actions including effects on the mitochondria^5^. MMPs have even been shown to reside in mitochondrial membranes^5^.

We have demonstrated in our lab that MMPs play a role in injury at the time of cold preservation. In both our animal model and human clinical analyses, there was a considerable release of MMP-2 and MMP-9 as well as injury markers LDH and NGAL^6,7^ into the preservation solution used in machine cold perfusion (perfusate). We have data that showed that the addition of MMP inhibitors doxycycline (Doxy) or MMP-2 siRNA led to a significant decrease in MMP-2 and MMP-9 and injury markers^10^. Similar results implicating MMPs in cardiac injury and the protective effect of Doxy were also shown in our lab^8,9^.

It has been shown that the accumulation of succinate is a universal metabolic signature of ischemia, and that this drives the accumulation of reactive oxygen species in the mitochondria during reperfusion^10^. That the mitochondria are central to ischemia and/or ischemia–reperfusion injury is further supported by work of other groups looking at the protective effects of hydrogen sulfide and carbon monoxide. The mechanism of these interventions appears to be the induction of a hibernation-like state in organs being preserved for the purposes of transplantation^11,12^. Using a proteomic approach, our group showed that several enzymes involved in glycolysis were increased when kidneys were cold perfused with solution containing Doxy, suggesting that the protective mechanism of Doxy might be as a result of increased glycolysis in addition to preservation of mitochondrial structure and function^13^.

We hypothesize that the MMP inhibitor Doxy, a benign and inexpensive clinically used antibiotic that has been shown to protect from *cold* ischemic injury, also protects the kidney from *warm* ischemic injury when given preemptively, before cardiac arrest in a DCDD animal model.

## Materials and methods

### Treatment and control arms (Fig. 1)

**Figure 1:**
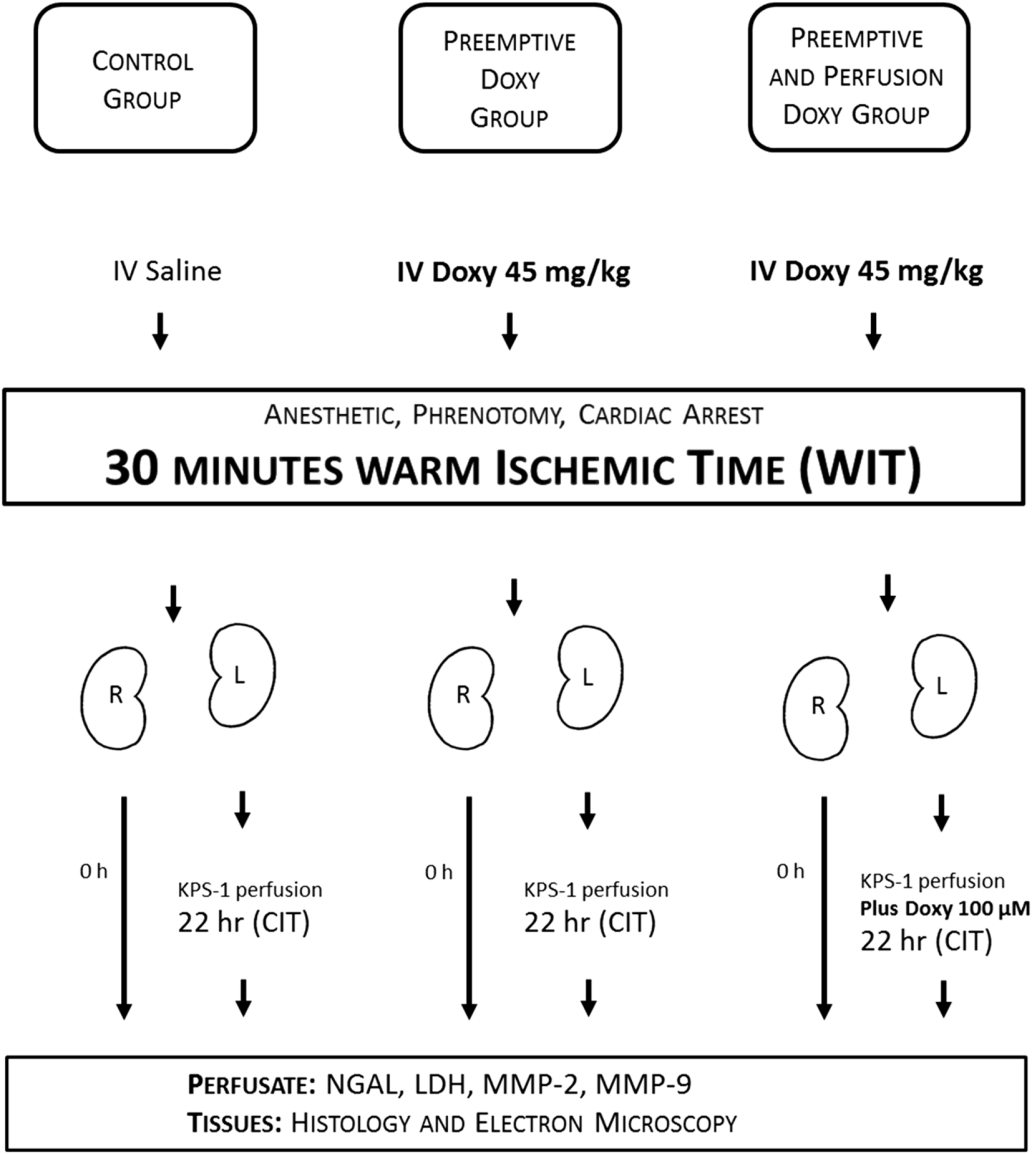
Study design, showing details of each of the 5 groups [2 right kidney groups to assess the effect of Doxy on *warm* ischemic injury (that is, with or without pre-arrest Doxy), and 3 left kidney groups to assess the effect of Doxy on *warm and cold* ischemic injury]. CIT: cold ischemic time, Doxy: doxycycline, KPS-1: Kidney Perfusion Solution-1, WIT: warm ischemic time.

Eighteen male Sprague–Dawley rats (n = 6 per group) weighing 200–250 g (Charles River, Burlington) were randomly assigned to the 3 groups. Rats in the treatment groups received the MMP inhibitor Doxy (45 mg/kg) intravenously 30 min prior to laparotomy (‘Preemptive Doxy’ group) and/or had kidney perfusion for 22 h with KPS-1 Perfusion Solution containing Doxy (100 μM) (‘Preemptive and Perfusion Doxy’ group). Doxy doses were determined from previous studies in our lab^9^. Rats in the control arm received saline intravenously and underwent kidney perfusion with KPS-1 solution without Doxy. A sample size of approximately 6 rats per arm has been standard in these types of studies over the last decade in our lab; in this study, each rat contributes two kidneys, each to a different ‘group’.

### DCDD donation model

Animals were anesthetized with Isofluorane and ventilated. The abdomen was opened with a midline incision, and cardiac arrest was induced by phrenotomy, as previously described^14,15^. After 30 min of WI after cardiac arrest to ensure ample injury to the kidneys, the left kidney was exposed and the left renal artery encircled and cannulated in situ.

### Flushing and machine cold perfusion

The left kidney was removed and flushed with Kidney Preservation Solution-1 (KPS-1, 20 cc over one hour) at 4C to flush out the blood. Each cannulated left kidney was then suspended in a 50 cc tube (Cole Parmer, Vernon Hill, IL) containing 20 cc of with KPS-1 with or without Doxy (100 μM), depending on the group. The perfusion was carried out for 22 h in a 4 °C cold room using a micro-pump (Gilson, Middleton, WI) at a flow rate of approximately 15–25 cc per hour, titrated to pressures of 30 mmHg, not to exceed 40 mmHg. The recirculating perfusate was collected at 22 h and quick frozen at − 80 °C for batched biochemical and zymographic analysis. A portion of each kidney was placed formalin in preparation for light microscopy and another in glutaraldehyde in preparation for electron microscopy.

### Analysis of perfusate from machine cold perfusion

The perfusates were analyzed for MMP-2 and MMP-9 activity, LDH, and NGAL collected at 22 h, as per our previous studies. Gelatin zymography for MMP-2 and MMP-9 activity was performed using the protocol of Heussen and Dowdel^10^. LDH activity was measured by LDH activity assay (Sigma-Aldrich, St. Louis, MO, USA). NGAL was measured using a commercially available ELISA kit (Abcam, Toronto, Canada).

### Assessment of histological injury

Slides were prepared and stained with Hematoxylin and Eosin (H&E) staining and reviewed by a renal pathologist (TB). Each was scored using the EGTI (Endothelial, Glomerular, Tubular, and Interstitial) scoring system for rat kidneys as described by Khalid et al.^16^.

### Electron microscopy

One-millimeter cubes of kidney tissues were fixed with glutaraldehyde then postfixed in osmium tetroxide, dehydrated in graded ethanol, and embedded in epoxy resin. Ultra-thick sections were cut and stained with uranyl acetate and lead citrate and random areas photographed using digital transmission EM, ensuring tubules were included. Mitochondria from five fields (at 5000× magnification) from each group were scored according to the Flameng grading for mitochondrial injury^17^. Briefly, Flameng grading involves using specific criteria to assign a score from 0 to 4, with higher scores indicating more injury, to each mitochondrion. Flameng scores have been shown to correlate with ischemic time and biochemical markers in the clinical setting^18^.

### Statistical analysis

Comparisons between groups were carried out using SPSS 25 (Chicago, USA). The Mann–Whitney U test was used for comparisons involving scoring system results and the Student’s t-test was used for all other results. A *p* value of < 0.05 was taken as significant in all cases.

### Ethics statement

This study conforms to the Guide to the Care and Use of Experimental Animals of the Canadian Council on Animal Care. The study was reviewed and approved by the University of Saskatchewan Biomedical Research Ethics Board (BioREB #20130073 amended 2017).

## Results

### Injury biomarkers

**Figure 2:**
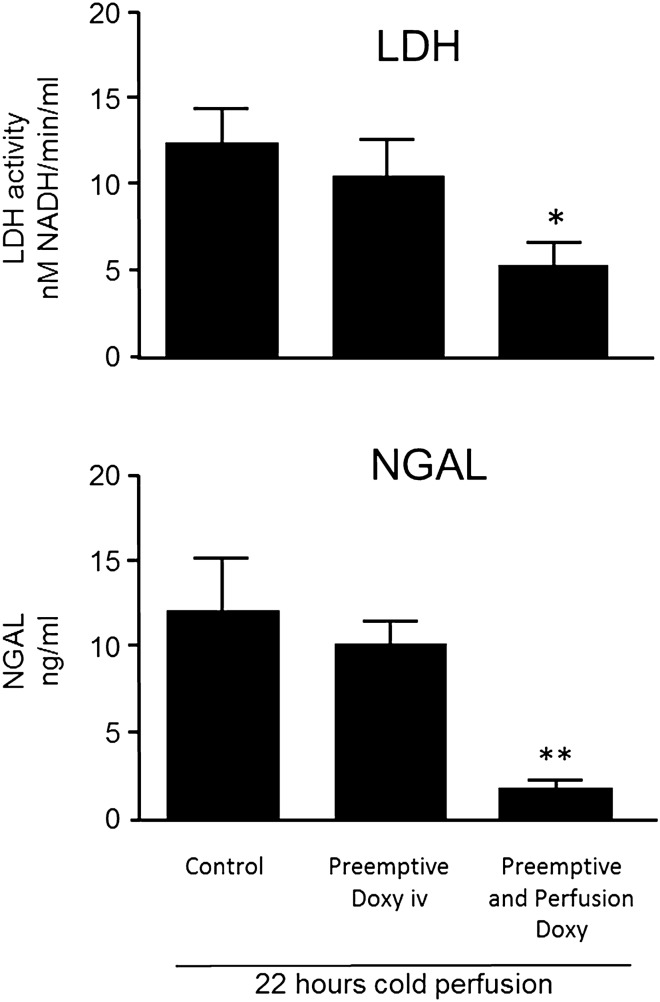
LDH and NGAL in perfusates from left kidneys after 22 h of machine cold perfusion at 4 °C. * *p* < 0.05, **p < 0.01, compared to Control group.

*NGAL and LDH* (Fig. 2): NGAL in the perfusate of the ‘Preemptive and Perfusion’ group was significantly decreased (1.8 ± 0.4 ng/mL, *p* = 0.007) compared to the Control group (12.1 ± 3.1 ng/mL), while NGAL for the ‘Preemptive Doxy’ group was not significantly different (10.1 ± 1.4 ng/mL, *p* = 0.42). LDH, a less specific marker of injury, showed a similar pattern.

### *MMP-2 and MMP-9 in perfusate* (Fig. 3)

**Figure 3:**
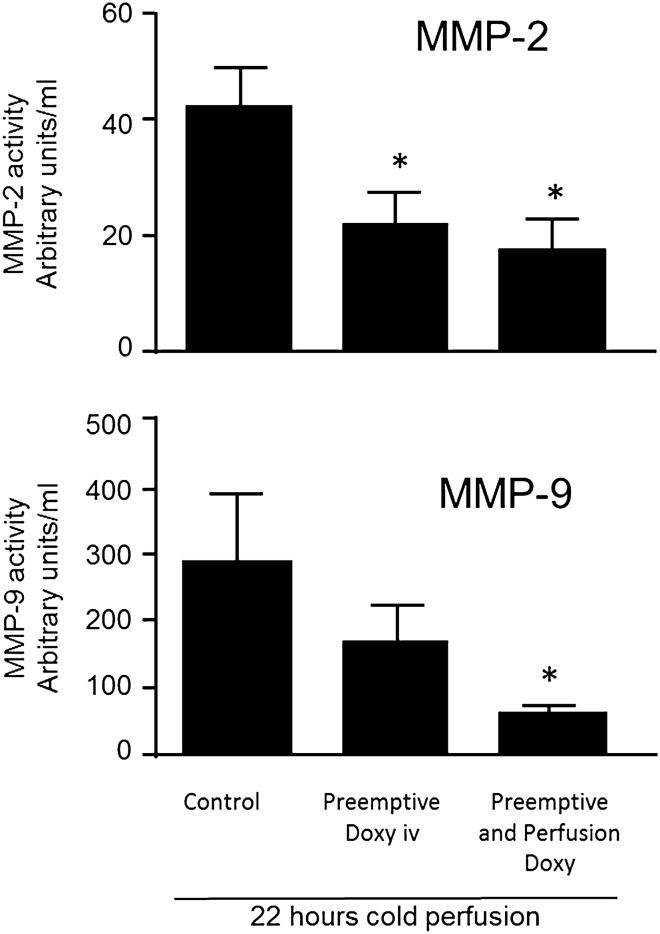
MMP-2 and MMP-9 in perfusates from left kidneys after 22 h of machine cold perfusion at 4 °C. *p < 0.05 compared to control group.

MMP-2 in the perfusate was significantly reduced in both treatment groups [Control 43.7 ± 7.2 arbitrary units, versus Preemptive Doxy group 23.2 ± 5.5 (*p* = 0.03), and ‘Preemptive and Perfusion’ group 18.0 ± 5.6 arbitrary units (*p* = 0.02)]. A statistically significant decrease in MMP-9 in the perfusate was seen for the ‘Preemptive and perfusion’ group (65.3 ± 17.5 arbitrary units versus Control 291.0 ± 97.2 arbitrary units, *p* = 0.03). Mean MMP-9 in perfusate was 173.8 ± 52.5 arbitrary units for the Preemptive group (*p* = 0.16).

### *Light microscopy/histology* (Fig. 4)

**Figure 4:**
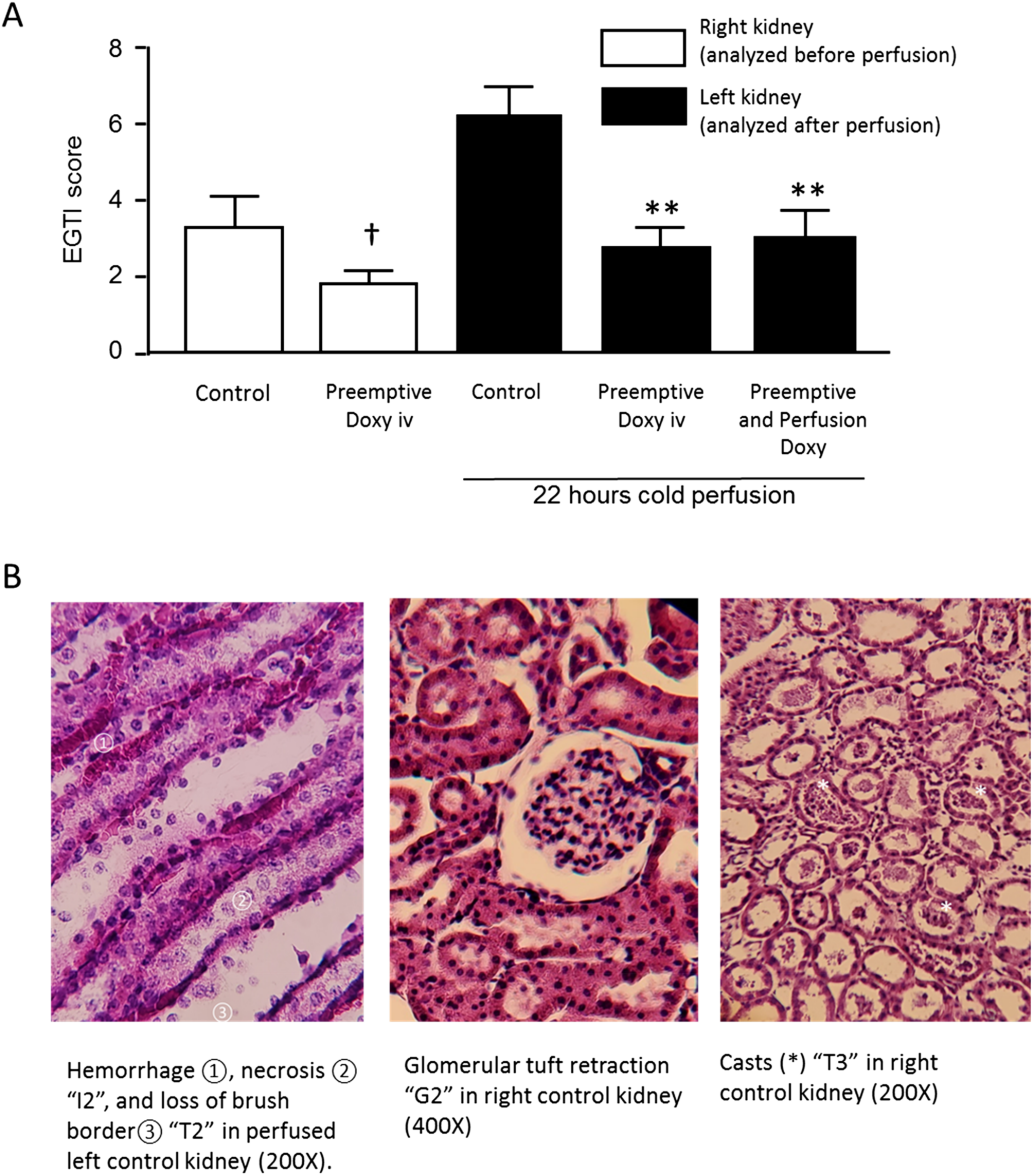
(**A**) Histological injury as measured by the EGTI (Endothelial, Glomerular, Tubular, and Interstitial) score of Khalid et al. *p < 0.01 compared to the right kidney (Warm Ischemia) Control Group, using the Mann–Whitney U test. †*p* = 0.105 compared to the left kidney (Warm and Cold Ischemia) Control Group, using the Mann–Whitney U test. (**B**) Examples of EGTI scoring.

The DCDD model produced significant injury in all kidneys. There was a trend towards improvement in EGTI injury scores in the right kidneys, from intravenous preemptive Doxy administered prior to warm ischemia (1.8 ± 0.3 versus Control 3.3 ± 0.8, *p* = 0.105). After perfusion of the left kidneys, injury scores were significantly improved in the ‘Preemptive Doxy’ group (3.0 ± 0.4, *p* = 0.004) and the ‘Preemptive and perfusion Doxy’ group (3.3 ± 0.5, *p* = 0.009) versus the Control group (6.4 ± 0.8).

### Electron microscopy

**Figure 5:**
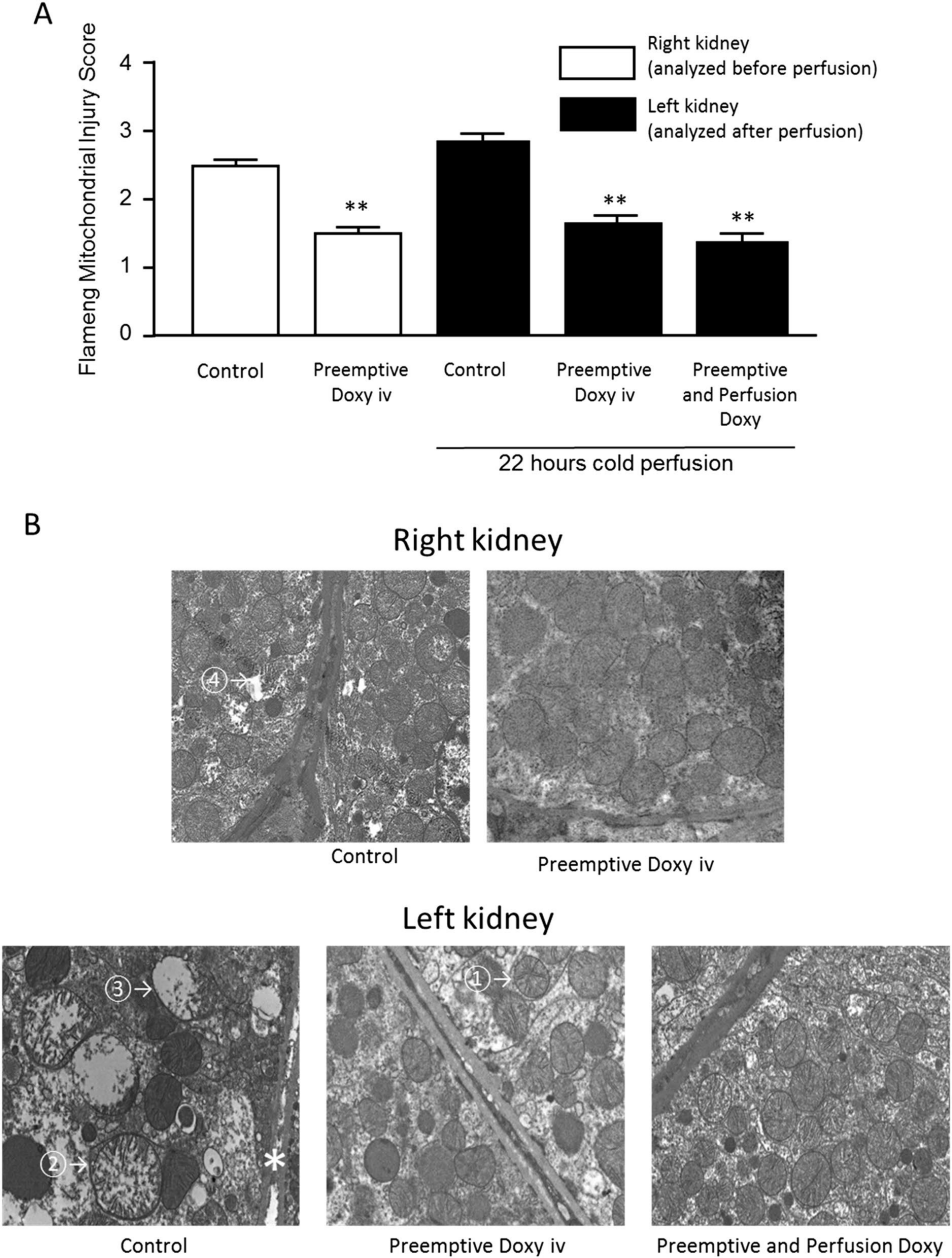
(**A**) Mitochondrial injury in Donation after Circulatory Death rat kidneys as measured by the Flameng Mitochondrial Injury Score. ** *p* < 0.01 compared to Control group, using the Mann–Whitney U test. (**B**) Representative electron micrographs of control and treatment group kidneys with examples of Flameng mitochondrial scoring (5000 × magnification). (1) Normal structure with granules absent. (2) Swollen mitochondria with clarification of the matrix. (3) Disruption of mitochondrial crests. (4) Loss of integrity of the mitochondrial inner and outer membrane. (*) Endothelial detachment.

The Flameng mitochondrial injury score (Fig. 5A) in the right kidneys (exposed to warm ischemia alone) was lower in the ‘Preemptive Doxy’ group (1.54 ± 0.05, *p* < 0.01) compared to the Control group (2.5 ± 0.07). In the left kidneys, exposed to warm and cold ischemia, the injury score was highest for the Control group mitochondria (2.88 ± 0.35) and significantly lower in the treatment groups (‘Preemptive Doxy’ 1.64 ± 0.41, *p* < 0.001 and ‘Preemptive and perfusion Doxy’ 1.41 ± 0.37, *p* < 0.001), with the difference between the latter two groups being significant as well (*p* = 0.04).

There were subjective differences in the appearance of the extracellular matrix, with endothelial detachment seen in the Control groups (Fig. 5B).

## Discussion

These results suggest that Doxy, given before cardiac arrest and WI, was associated with a reduction in injury markers and cellular and mitochondrial injury morphology in a rat model of DCDD kidney preservation.

Although our study was designed to assess the effect of Doxy in protecting transplant kidneys, MMPs are ubiquitous, and our laboratory has indeed documented protective effects of Doxy on ischemic cardiac injury. Our results should therefore be of relevance to the study of non-transplant ischemia–reperfusion phenomena, in cases where the ischemic event is planned or anticipated, such as occlusion of the renal artery during partial nephrectomy. Furthermore, similar protective effects should be expected for the other solid organs procured at the time of DCDD.

We have previously shown that Doxy helps protect against cold ischemic injury at the time of machine cold perfusion^9^. The current results suggest that injury may also be prevented by administering Doxy prior to warm ischemic injury. For some of the injury measures, there was a significant incremental protection from the use of preemptive Doxy and Doxy in the perfusion fluid, while for other measures, there was not. An incremental effect was seen in terms of specific proteins released into the perfusate. Although we have shown that the proteins released into the perfusate do indeed reflect kidney injury during preservation, this does not necessarily reflect the intracellular state of the cells. There is increasing evidence to suggest that the structure and enzymatic integrity of the mitochondria may be the key to the cell surviving preservation or ischemia–reperfusion^10^ and that accumulation of succinate may be central to ischemia-reperfusion injury.

The mechanism whereby Doxy, through inhibition of MMP-2, protects the kidney in the setting of *cold* ischemia appears to be through the protection of mitochondria as well as the preservation of the ECM. A recent publication from our lab used a proteomics approach to identify the intracellular enzymes that were affected by Doxy in this same rat model^13^. We identified 8 intracellular enzymes, of which the majority are involved in glycolysis and in mitochondrial function. The results in this current study support that a similar benefit, and possibly a similar mechanism, contributes to protection of kidneys from *warm* ischemia by Doxy. The administration of the MMP inhibitor before ischemia potentially prepares the cells or their mitochondria to better tolerate the warm ischemic insult, in contrast to administering the drug after the warm ischemic insult has already occurred.

Kunugi et al. examined the role of MMPs in renal warm ischemic injury in a study where the renal artery was clamped for 30–120 min, and then the kidney was allowed to reperfuse by removing the clamp^19^. The degree of acute tubular injury, necrosis, and apoptosis was markedly less in MMP-2 deficient transgenic mice, suggesting an important role for MMP-2 in warm ischemic injury. Our results agree with Kunugi’s in terms of the importance of MMP-2 in injury despite the slightly different injury mechanism.

Our study, like many studies involving an animal model and several treatment arms, is limited by a relatively small sample size.

It has been noted that our model that includes a warm ischemia time of 30 min seems extreme compared to the clinical scenario of five minutes of “no touch” time followed by about another five minutes before cannulation and cooling. We chose a warm ischemia time of 30 min because we wanted to ensure that there was considerable injury so that an intervention that helped might be more likely to show a significant benefit. We also wanted to account for injury that occurs clinically during the hypotension and relative warm ischemia of the agonal phase (between withdrawal of life support and cardiac arrest). In our model, the agonal phase was relatively short, between 8 and 12 min in all cases.

Finally, the protective effect of Doxy on kidneys destined for transplant should ideally be demonstrated with a model that involves transplantation of the treated and untreated kidneys in an animal model. Our current study was a pilot/proof of concept on a small research grant. Kidney transplantation in a rat model requires extensive training and considerable expertise in microsurgery and remains a procedure done by only a handful of ‘experts’ worldwide. Our centre did not possess such expertise; however, we have plans to collaborate with a larger centre to test Doxy's preemptive protective effect in a rat transplant model.

From a clinical point of view, the administration of Doxy prior to the withdrawal of life-supporting treatments is a potential intervention that is simple and non-toxic. In cases of DCDD donation, one must always be wary of the administration of drugs that might be perceived as bringing harm to the donor. It seems unlikely that the administration of this antibiotic would lead to harm in the donor, except on the very rare occasion that the donor has a severe allergy to Doxy. Given the separation of the donation team and the transplant team at most centers, it might be challenging to gain acceptance from the donation team for an intervention that only benefits the recipient or recipients of the donor’s organs. Still, the administration of Doxy prior to cardiac arrest would undoubtedly be of a lower risk than the administration of intravenous heparin in high doses, a common practice in many centres that participate in DCDD donation.

There is one potential challenge that bears mentioning. The administration of Doxy prior to cardiac arrest might help improve the quality of not only the kidneys, but the heart, lung, liver, pancreas, and small bowel as well. As a result, there is the potential that the administration of Doxy prior to arrest may protect the heart, and may prolong the time to cardiac arrest to beyond the two hours most programs consider an acceptable time to wait for asystole. This is potentially a challenge for any pre-cardiac arrest intervention prior to DCDD and something that will need to be discussed in the future. In our study, we did not observe a significant difference in time to cardiac arrest between the groups that received preemptive Doxy and those that did not. It may be that asystole and the preservation of cardiomyocytes are independent of each other, with asystole occurring instead as a result of electrolyte abnormalities.

In conclusion, our pilot study in an animal model suggests that the preemptive administration of Doxy prior to the withdrawal of life support and planned DCDD organ procurement might be able to protect transplant kidneys (and other organs) from both warm ischemic and cold ischemic injury. In the long term, this has the potential to increase the quality of transplant kidneys after DCDD and indirectly increase the number of donor organs available.

## Abbreviations

CI: Cold ischemia
DCDD: Donation after circulatory determination of death
DNDD: Donation after neurological determination of death
Doxy: Doxycycline
ECM: Extracellular matrix
KPS-1: Kidney Perfusion Solution-1
WI: Warm ischemia

## References

[1] Moser M (2012). Five-year experience with donation after cardiac death kidney transplantation in a Canadian transplant program: Factors affecting outcomes. CUAJ.

[2] Marek C (2014). The prognostic value of time needed on dialysis in patients with delayed graft function. Nephrol. Dial. Transpl..

[3] Saidi RF (2007). Outcome of kidney transplantation using expanded criteria donors and donation after cardiac death kidneys: Realities and costs. Am. J. Transpl..

[4] Hooper NM (1994). Families of zinc metalloproteinases. FEBS Lett..

[5] Hughes BG, Fan X, Cho W, Schulz R (2014). MMP-2 is localized to the mitochondria-associated membrane of the heart. Am. J. Physiol. Heart Circ. Physiol..

[6] Moser M, Sawicka K, Arcand S, O’brien P, Luke P (2017). Proteomic analysis of perfusate from machine cold perfusion of transplant kidneys: Insights into protection from injury. Ann. Transpl..

[7] Moser M (2016). Protection of the transplant kidney from preservation injury by inhibition of matrix metalloproteinases. PLoS ONE.

[8] Cheung PY (2000). Matrix Metalloproteinase-2 contributes to ischemia-reperfusion injury in the heart. Circulation.

[9] Fert-Bober J (2008). Inhibiting matrix metalloproteinase-2 reduces protein release into coronary effluent from isolated rat hearts during ischemia-reperfusion. Basic Res. Cardiol..

[10] Chouchani ET (2014). Ischaemic accumulation of succinate controls reperfusion injury through mitochondrial ROS. Nature.

[11] Sener A (2013). Carbon monoxide releasing molecules inhibit cell death resulting from renal transplantation related stress. J. Urol..

[12] Juriasingani S, Akbari M, Chan JY, Whiteman M, Sener A (2008). H2S supplementation: A novel method for successful organ preservation at subnormothermic temperatures. Nitric Oxide.

[13] Moser M (2020). Protection of the transplant kidney during cold perfusion with doxycycline: Proteomic analysis in a rat model. Proteome Sci..

[14] Kearns MJ (2017). A rodent model of cardiac donation after circulatory death and novel biomarkers of cardiac viability during ex vivo heart perfusion. Transplantation.

[15] Li J (2017). Optimized rat models of donation after cardiac death for bench to bed translation. Int. J. Clin. Exp. Med..

[16] Khalid U (2016). Kidney ischaemia reperfusion injury in the rat: The EGTI scoring system as a valid and reliable tool for histological assessment. J. Histol. Histopathol..

[17] Flameng W, Borgers M, Daenen W, Stalpaert G (1980). Ultrastructural and cytochemical correlates of myocardial protection by cardiac hypothermia in man. J. Thorac. Cardiovasc. Surg..

[18] Li L (2018). Pathological features of mitochondrial ultrastructure predict susceptibility to post-TIPS hepatic encephalopathy. Can. J. Gastroenterol. Hepatol..

[19] Kunugi S (2011). Inhibition of matrix metalloproteinases reduces ischemia-reperfusion acute kidney injury. Lab. Invest..

